# The effect of argon cold atmospheric plasma on the metabolism and demineralization of oral plaque biofilms

**DOI:** 10.3389/fcimb.2023.1116021

**Published:** 2023-03-09

**Authors:** Haowei Zhao, Xu Wang, Zhuo Liu, Ye Wang, Ling Zou, Yu Chen, Qi Han

**Affiliations:** ^1^ State Key Laboratory of Oral Diseases and National Clinical Research Center for Oral Diseases, Department of Oral Pathology, West China Hospital of Stomatology, Sichuan University, Chengdu, China; ^2^ School of Mechanical Engineering, Sichuan University, Chengdu, China; ^3^ College of Intelligent Systems Science and Engineering, Huber Minzu University, Enshi, China; ^4^ State Key Laboratory of Oral Diseases and National Clinical Research Center for Oral Diseases, Department of Endodontics, West China Hospital of Stomatology, Sichuan University, Chengdu, China

**Keywords:** argon, cold atmospheric plasma, oral plaque biofilms, enamel, demineralization

## Abstract

**Objective:**

The aim of this study was to design and optimize a cold atmospheric plasma (CAP) device that could be applied in an oral environment and to study its effects on plaque biofilm metabolism and regrowth, as well as microbial flora composition and enamel demineralization.

**Method:**

CAP was obtained through a dielectric barrier discharge device; the optical properties were analyzed using emission spectroscopy. The electrochemical analysis of plasma devices includes voltametric characteristic curves and Lissajous. The *Streptococcus mutans* (UA159) and saliva biofilms were treated *in vitro*, and the effects of CAP on biofilm metabolism were investigated using MTT and lactate dehydrogenase assays. The duration of antibacterial activity on biofilms was examined, scanning electron microscopy was used to observe the morphology of biofilms, and 16S rRNA sequencing was used to explore the influence of CAP on the microbial flora composition of saliva biofilms. An *in vitro* model of biofilm-enamel demineralization was designed, and the effect of CAP on enamel demineralization was evaluated by micro surface hardness and micro-CT analysis.

**Results:**

CAP had antibacterial proliferative ability toward *Streptococcus mutans* biofilms and saliva biofilms and was stronger than ultraviolet under the same tested conditions. After 24 h, the antibacterial effect disappeared, which proved the short-term timeliness of its bactericidal ability. CAP can inhibit the acid production of biofilms, and its inhibitory effect on saliva biofilms can be extended to 24 h. CAP had a strong ability to regulate the composition of plaque biofilms, especially for *Lactococcus* proliferation, a major acid-producing bacterium in microcosm biofilms. The CAP-treated enamels were more acid-tolerant than non-treated controls.

**Conclusion:**

CAP had an explicit bactericidal effect on caries-related biofilms, which is a short-term antibacterial effect. It can inhibit the acid production of biofilms and has a downregulation effect on *Lactococcus* in saliva biofilms. CAP can help reduce demineralization of enamel.

## 1 Introduction

The oral cavity is a diverse microecological environment in which more than 700 microorganisms reside (Kitamoto et al., 2020). The imbalance of flora can lead to the proliferation of oral conditional pathogenic bacteria, which can cause the development of infectious diseases (Li et al., 2021a). Dental caries is this chronic endogenous infection caused by the proliferation of oral commensal flora, which is manifested by the proliferation of conditionally pathogenic bacteria contributing to the acidification of the microenvironment, resulting in an increase in the ratio of acid-producing and acid-tolerant bacteria(Bowen et al., 2018). Further acidification of the oral microenvironment disrupts the balance of mineralization and remineralization of the dental surface and disturbs calcium and phosphorus metabolism in the hard tissues, resulting in loss of inorganic material and destruction of organic collagen (Pandya and Diekwisch, 2019). Therefore, regulating the balance of flora, controlling the proliferation of conditionally pathogenic bacteria, and avoiding the acidification of the microenvironment are the key factors to reduce dental caries (Chen et al., 2020). Moreover, the high incidence of dental caries has a great impact on public health resources, thus regulating the flora balance, controlling the proliferation of conditionally pathogenic bacteria, and avoiding the acidification of the microenvironment also play a benign role in promoting public health resources (Peres et al., 2019).

*Streptococcus mutans (S. mutans)* has always been a highly detectable organism in the oral cavity of people with high risk of dental caries and is still considered as one of the main caries-causing organisms (Ramadugu et al., 2021; Zhang et al., 2022). Generally, the cariogenicity of *S. mutans* controlled by exogenous drugs is indirectly confirmed by the amount of bioaugmentation and metabolic acid production. Although the current demineralization-related tests do not fully reflect the degree of caries progression (Shahmoradi and Swain, 2017; Wang et al., 2019), the use of demineralization tests to evaluate the degree of caries progression is a valid evaluation method compared with the previously mentioned methods.

Exogenous antimicrobial drugs have always been an important part of the clinical approach to infectious diseases in dentistry, and traditional antimicrobial drugs have many undesirable side effects in use (Liang et al., 2020), making new antimicrobial modalities a research challenge and a hot topic in this field. Traditional oral antimicrobial drugs are mostly targeted at the bacterial envelope (Li et al., 2021b), increasing the permeability of the cell membrane, leading to leakage of intracellular material or penetration into the cell, and causing intracellular dehydration or denaturation of cellular proteins, such as chlorhexidine, alcohol, or camphor (Wikén Albertsson et al., 2013). However, the residual problems associated with the use of traditional oral antimicrobial drugs have led to many limitations in clinical application, such as tooth staining, unpleasant taste, occasional allergic reactions, risk of oral microbial tolerance, cytotoxicity, and contraindications in pregnant and lactating patients (Nakanishi et al., 2018). Therefore, the development of new residue-free antimicrobial oral disinfection methods is of great clinical importance.

Plasma is the fourth state of matter and was discovered by Sir William Crookes in 1879 (Borges et al., 2021). Plasma can be divided into thermal plasma and cold atmospheric plasma (CAP) (Von Keudell and Schulz-Von Der Gathen, 2017). The source gases of CAP include helium, argon, nitrogen, helium oxygen (mixture of helium and oxygen), and air. CAP has a wide range of biomedical applications, such as modifying materials and promoting skin wound healing (Guo et al., 2022). Currently, CAP has started to be used in dentistry, such as surface modification of implant materials and tooth whitening (Khaledian et al., 2019; Nam et al., 2021). However, there are relatively few studies on whether CAP can control the acid-producing metabolism of oral biofilm to achieve a regulatory effect on oral microorganisms; secondly, studies on whether argon cryogenic plasma could inhibit the proliferation and metabolism of caries-causing or acid-producing microorganisms and affect tooth demineralization have not been reported. To this end, a low-temperature atmospheric plasma device was designed in this paper to investigate its effects on the proliferation of oral microorganisms and demineralization of tooth enamel.

## 2 Materials and method

### 2.1 Plasma device

**Figure 1:**
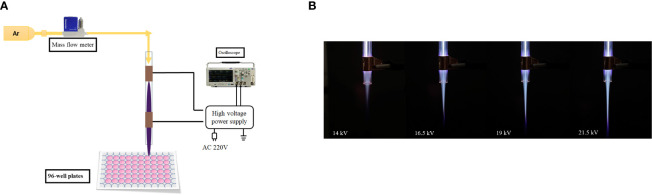
**(A)** Dielectric-barrier discharge plasma device. **(B)** Plasma appearance under different voltages.

An argon dielectric-barrier discharge (DBD) device equipped with double-ring electrodes was used, as shown in the **Figure 1A**. The plasma device consisted of gas supply system, mass flow controller, gas channel, and high-voltage AC power supply. The dielectric material (gas channel) was a quartz tube with an inner diameter of 4 mm, an outer diameter of 6 mm, and a length of 100 mm. The electrode was copper conductive tape with width of 8 mm. The distance between the low-voltage electrode and the outlet of the quartz tube was 8 mm. The center distance between the low-voltage electrode and the high-voltage electrode was 15 mm. Argon entered from the top of the quartz tube, and the purple plasma was ejected under the action of air flow, as shown in **Figure 1B**. The distance between the quartz tube outlet and targets was adjusted to 2 cm. The peak voltage was 19 kV, and gas flow was 2 l/min.

### 2.2 Electrical and spectral analyses

The volt–ampere characteristic curve, collected using an oscilloscope, can directly reflect the variation of the voltage and current of DBD. A Lissajous figure was obtained for calculating the discharge power. The optical characteristics of CAP were analyzed by the emission spectrum technique. The major group species in the process of discharge were deduced according to the position of the optical emission line.

### 2.3 Biofilm formation

#### 2.3.1 *S. mutans* biofilms

**Figure 2:**
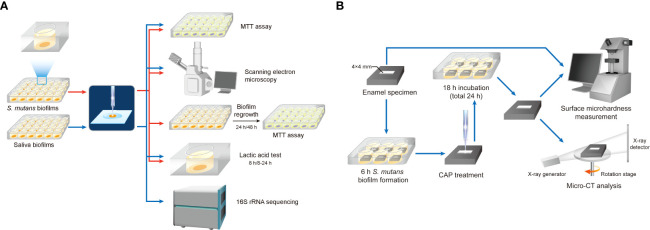
Workflow diagram. **(A)** Effects of CAP on the proliferation of oral microorganisms. **(B)** Effects of CAP on demineralization of tooth enamel.

*S. mutans* (UA159) was used in this study. The bacteria were diluted with a final concentration of 1× 10^8^ CFU/ml in brain heart infusion (BHI, Oxoid) media. For biofilm growth, 1% sucrose was added to BHI media and *S. mutans* was inoculated. Sterilized glass disks were placed into a 24-well plate. Then, 1.5 ml inoculum was added to each well and incubated under anaerobic conditions (37°C, 5% CO_2_). The disks were transferred into a new 24-well plate with fresh media after 8 h of incubation. After 24 h of incubation, the disks were washed with PBS before treatment. The workflow is shown in **Figure 2**.

#### 2.3.2 Saliva biofilms

Saliva was collected from six healthy adult donors, who had natural dentition but no periodontal disease, had no active caries, and were not taking antibiotics in the past 3 months. Donors were instructed not to brush their teeth for 24 h or intake food or drink for 2 h before saliva donation. Non-stimulating saliva was collected and kept on ice. The saliva from each of the six donors were mixed and diluted in sterile glycerol to a concentration of 70%, then stored at −80°C.

The saliva-glycerol stock was added with 1: 50 final dilutions to SHI media as inoculum (Tian et al., 2010; Li et al., 2017). The SHI media contain proteose peptone 10 g/l, trypticase peptone (Oxoid) 5.0 g/l, yeast extract (Oxoid) 5.0 g/l, KCl 2.5 g/l, sucrose 5 g/l, hemin 5 mg/l, VitK 1 mg/l, urea 0.06 g/l, arginine 0.174 g/l, mucin (type II, Sigma) 2.5 g/l, sheep blood 5%, and N-acetylmuramic acid (Sigma) 10 mg/l. Sterilized glass disks were placed into a 24-well plate, and 1.5 ml of inoculum was added to each well, incubated under anaerobic conditions (37°C, 5%CO_2_) for 8 h. Then, disks were transferred to new 24-well plates with fresh SHI media and incubated for another 16 h, for a total of 24 h.

### 2.4 MTT assay

The MTT assay is based on the cleavage of the yellow-colored tetrazolium salt, 3-(4,5-dimethylthiazol-2-yl)-2,5-diphenyl tetrazolium bromide, into a blue-colored formazan by the mitochondrial enzyme succinate-dehydrogenase^[25]^. Glass disks with 24-h biofilms were transferred to a new 24-well plate. The MTT solution (0.5 mg/ml MTT in PBS, BioFroxx, Germany) was added and followed by a 2-h incubation at 37°C in 5% CO_2_. Metabolically active bacteria reduced the MTT to formazan. Disks were transferred to a new 24-well plate containing 1 ml of dimethyl sulfoxide (DMSO) to solubilize the formazan. The plate was placed on a gyratory shake for 20 min in the dark. The absorbance at 540 nm was measured *via* a microplate reader (SpectraMax M5). Experiments were performed in triplicates, and each group had three biofilm samples.

### 2.5 Biofilm image observation

The architecture of biofilms after CAP treatment was examined by scanning electron microscopy (SEM). Briefly, biofilms were fixed in 2.5% glutaraldehyde (v/v) at 4°C for 12 h then washed twice with PBS and dehydrated for 10 min using an increasing series of ethanol (30%, 50%, 70%, 80%, 85%, 90%, 95%, 100% (v/v)). Finally, biofilms were rinsed two times with hexamethyldisilane and coated with gold, followed by being observed after under SEM (Quanta 200, FEI, Hillsboro, OR, USA).

### 2.6 Biofilm regrowth

For biofilm regrowth, the 24-h-treated *S. mutans* biofilms were transferred to fresh BHI media (1% sucrose); the refreshing media were as described above. Bacterial viability was detected by MTT assay after 24 and 48 h of regrowth.

### 2.7 Lactic acid production

The level of lactic acid produced by biofilms was quantified to indicate the metabolic activity of biofilms. After CAP or UV treatment, biofilms on disks were gently transferred to fresh media and incubated for 24 h. The media were refreshed after 8 h, and the spent media were collected. The lactic acid concentration in the spent media was measured using an enzymatic spectrophotometric method (Deng et al, 2009). The principle of this method is based on the enzymatic conversion of L-lactate to pyruvate with the concomitant conversion of NAD^+^ to NADH (Jiancheng, Nanjing, China). The absorbance at 530 nm was measured, and acid concentration was calculated based on the standard curve.

### 2.8 16S rRNA sequencing

The treated saliva biofilms were subjected to Personalbio (Shanghai, China), where DNA were extracted, amplified, and purified according to standard procedures and sequenced as described below. Briefly, total DNA were extracted using Omega M5635 Soil DNA Kit (USA) and stored at -20°C. For detecting the quantity and concentration of DNA, 0.8% agarose gel electrophoresis and a NanoDrop were performed. The highly variable regions (V3–V4) of the bacterial 16S rRNA gene with a length of around 468 bp were selected for sequencing. The forward and reverse primers for amplification were barcode+ ACTCCTACGGGAGGCAGCA and GGACTACHVGGGTWTCTAAT, respectively. Each 20-μl PCR reaction contains 1 μl template DNA, 5 μl 5× buffer, 0.25 μl FastPfu DNA Polymerase, 2 μl dNTPs, 1 μl of each primer, and 14.75 μl DDW. The thermal cycling conditions were composed of pre-denaturation at 98°C for 30 s, followed by 25 cycles of 98°C for 30 s, 50°C for 30 s, and 72 °C for 30 s, with a final extension cycle at 72°C for 5 min. The amplified products were extracted from 2% agarose gel electrophoresis and purified by AMPure XP beads (Beckman, USA). The qualified library was sequenced using the MiSeq v3 Reagent Kit (600 cycles) with a 2 × 250-bp base read profile.

### 2.9 Enamel specimen preparation and biofilm inoculation

Enamel blocks were obtained from bovine teeth and cut into sections measuring 8*8*5 mm. These specimens were stored in 0.1% thymol before use. After washing in an ultrasonic cleaner, enamel blocks were embedded with polymethylmethacrylate and coated with acid-tolerant nail varnish, leaving a window of 4*4 mm on the labial enamel surface (**Figure 2B**). The enamel surfaces were polished using #800, 1,000, 1,200, 1,500, 2,000, 3,000, and 50,000 silicon carbide paper (Struers) consecutively. The specimens were then sterilized using ultraviolet and placed into six-well plates. All animal experimental procedures were approved by the Animal Ethics Committee of West China Hospital of Sichuan University, China (No. WCHSIRB-D-2022-255). *S. mutans* was inoculated at a final concentration of 1 × 10^7^ CFU/ml in 7 ml of BHI media with 1% sucrose per well. After 8 h of incubation in 5% CO_2_ at 37°C, the specimen was treated with CAP for 1 or 3 min with the same parameters as described above. Ultraviolet light-C (UV-C) 254 nm was used for comparison. Specimens with no treatment were regarded as control. After treatment, enamel blocks were transferred to new six-well plates with fresh media and incubated for another 16 h. Enamel blocks were washed with flowing water for 30 min and rinsed with an ultrasonic machine for 30 min to remove the biofilms.

### 2.10 Surface microhardness measurement

The surface microhardness (SM) of each enamel block was measured before and after treatment, by using a microhardness tester (Struers) under a load of 50 g for 15 s. Five quadrilateral indentations, spaced 100 μm apart from each other, were made in the center of each enamel block, arranged in a straight line. Enamel blocks with baseline surface microhardness (SM0) >300 was selected for further experiment. The surface microhardness of enamel after 16 h of incubation (SM1) was measured to calculate the surface microhardness loss percentage (SML%): SML%=(SM0-SM1)/SM0*100%

### 2.11 Micro-CT analysis

The micro-CT (Scanco Medical) analysis was performed at 90 kV and 155 μA on dry specimens. 0.5-mm-thick aluminum was used for hardware beam-hardening correction. Each specimen was scanned at 500proj/180° resolution with 5-μm precision. Data were analyzed using Scanco Evaluation. Lesion depth (LD) was calculated as follows: with the increase in demineralization depth, the corresponding mineral density of enamel increases. When the mineral density tended to be constant, it reached the normal enamel area. This point was regarded as the end of the lesion, and LD can be measured. Each sample were calculated triplicates.

### 2.12 Statistical analysis

Data were analyzed using the least significance difference test. Statistical analysis was performed using SPSS Statistics V.25.0 (IBM Corporation, USA) and a *P*-value of <0.05 was considered statistically significant.

## 3 Results

### 3.1 Electrical and optical analyses

**Figure 3:**
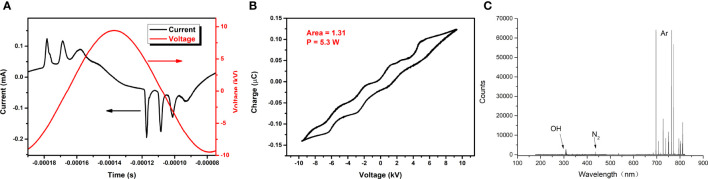
**(A)** Volt–ampere characteristic curve. **(B)** Lissajous diagram. **(C)** Diagram of optical emission spectroscopy.

When the applied voltage was 19 kV, volt–ampere characteristic curves in one cycle are shown in **Figure 3A**. The period was 116 µs, the peak voltage was 19 kV, the maximum current in the positive period was 0.12 mA, the waveform was a sine wave, and the phase difference was 90°.

When the applied voltage was 19 kV, the Lissajous figure of the plasma discharge device was as shown in **Figure 3B**. It was a closed parallelogram, and the power P of the discharge device can be calculated by the simplified formula P = *f CS*, where *f* stands for the frequency of the applied voltage, *C* represents the capacitance of the applied voltage, and *S* is the area of the Lissajous figure. The area in the figure was 1.31, *f* was 8.634 kHz, and *C* was 0.47 pF, so the power was 5.3 W.

When the applied voltage was 19 kV, the spectrum of CAP collected at 2 cm from the outlet of the quartz tube was as shown in **Figure 3C**: the most obvious peaks of the emission spectrum corresponded to Ar, hydroxyl radicals (-OH), and N_2_, respectively.

### 3.2 MTT assay

**Figure 4:**
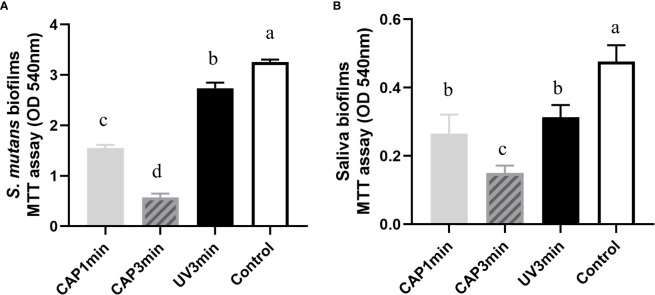
Comparison of the effects of CAP and UV on the biofilm activity. **(A)** *Streptococcus mutans* biofilm **(B)** saliva biofilm. Dissimilar letters indicate statistical differences between groups (*P* < 0.05).

The lower the absorbance of OD540 in the MTT test, the lower the amounts of viable bacteria. As shown in **Figure 4**, compared with the control group, exposure to CAP and UV caused a decrease in biofilm activity right after treatments; both *S. mutans* and saliva biofilms by CAP treatment resulted in a greater reduction in biofilms activity than by UV under the same tested conditions.

### 3.3 Scanning electron microscopy

**Figure 5:**
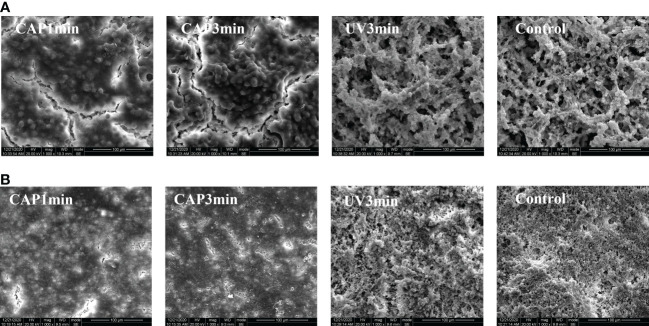
**(A)** SEM morphology observation of *Streptococcus mutans* biofilm (×1,000). **(B)** SEM morphology observation of saliva biofilm (×1,000).

**Figure 5** displays the representative biofilm images under SEM. Both *S. mutans* biofilms and saliva biofilms showed significant morphological alterations after CAP treatments when compared with the untreated controls, the contour of bacterial cells and biofilm structure vanished, whereas UV groups remained unchanged.

### 3.4 Biofilm regrowth

**Figure 6:**
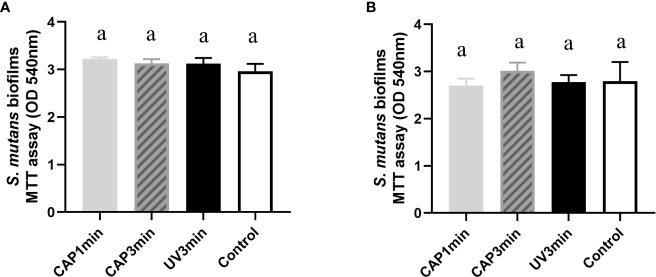
Bacterial activity after biofilm regrowth. **(A)** MTT value after 24 h of regrowth. **(B)** MTT value after 48 h of regrowth. Dissimilar letters indicate statistical differences between groups. There were no statistical differences between groups (*P* > 0.05).

No significant difference of bacterial proliferation was observed after 24 or 48 h biofilm regrowth, as shown in **Figure 6**, suggesting that CAP had a short-term antibacterial effect.

### 3.5 The effect on acid production of biofilms

**Figure 7:**
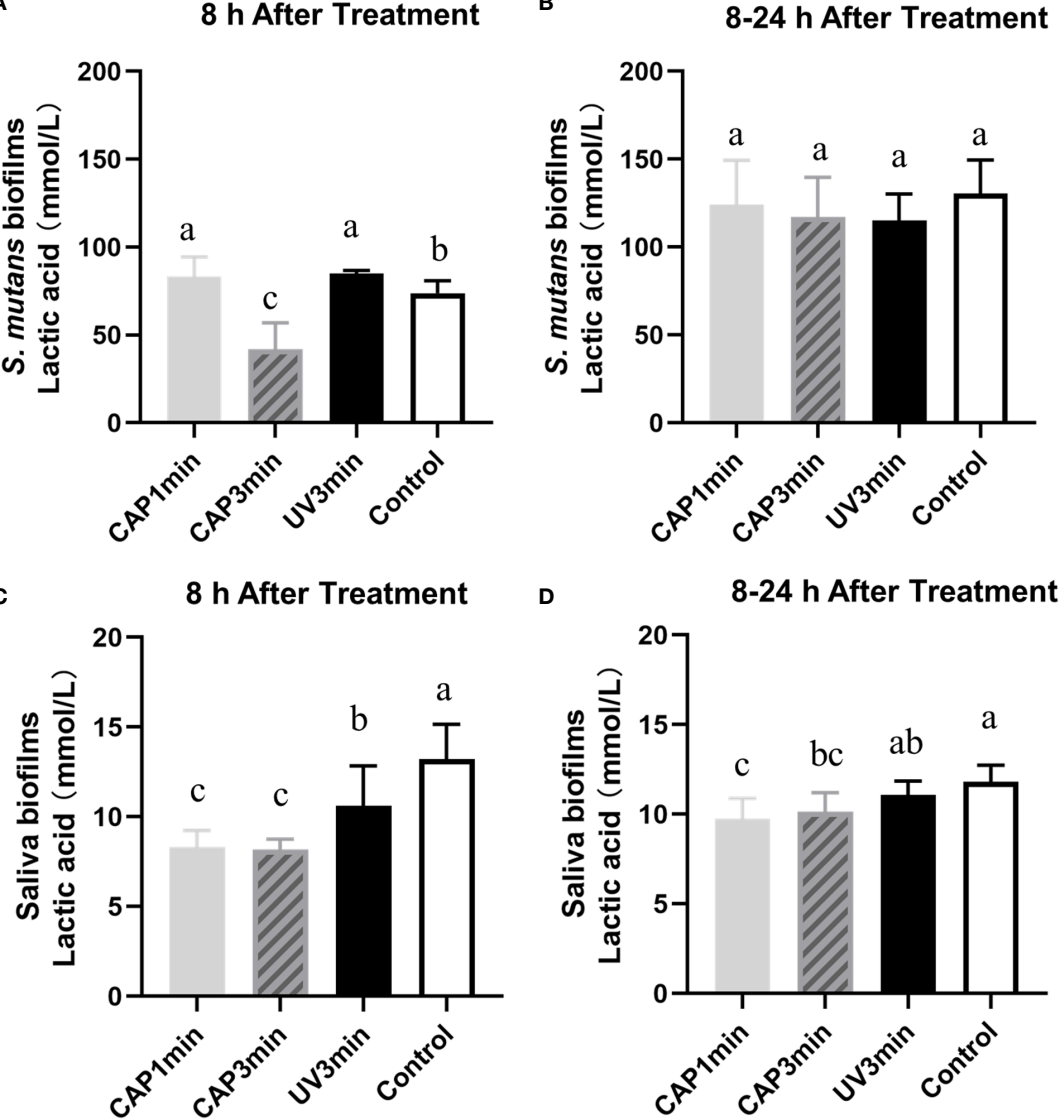
Lactic acid production of *Streptococcus mutans* biofilms **(A, B)** or saliva biofilms **(C, D)** at different times. A&C. The first 8 h after treatment. B&D. 8–24 h after treatment. Dissimilar letters indicate statistical differences between groups (*P* < 0.05).

Lactic acid production of biofilms are plotted in **Figure 7**. Results showed that CAP can inhibit the acid production of *S. mutans* biofilms and saliva biofilms. The lactic production of *S. mutans* biofilms dropped after CAP treatments and recovered at 24 h, whereas the saliva biofilms remained statistically lower at 24 h.

### 3.6 16S rRNA sequencing

**Figure 8:**
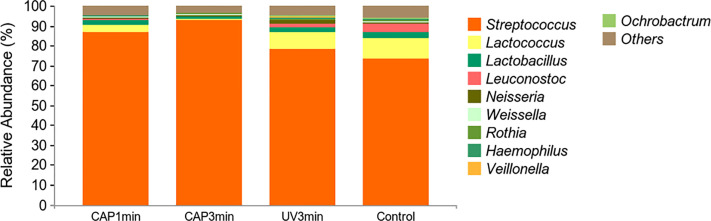
Changes of bacterial composition of saliva biofilms after CAP treatment. (Average relative abundance–genus).

**Figure 8** shows the average relative abundance of bacterial presence in saliva biofilms. At the genus level, the average relative abundance of *Streptococcus*, *Lactococcus*, *Lactobacillus*, *Leuconostoc*, *Neisseria*, *Weissella*, *Rothia*, *Haempophilus*, *Veillonella*, and *Ochrobactrum* were the top 10 in biofilms. The average relative abundance of *Lactococcus* decreased after CAP treatment, suggesting that CAP can repress the growth of low-pH *Lactococcus* species in saliva biofilms.

### 3.7 Surface microhardness measurement

**Figure 9:**
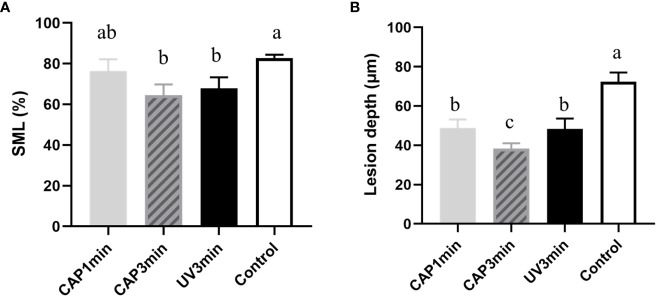
**(A)** Surface microhardness loss of enamel blocks after treatment followed by a 16-h acid attack. **(B)** Lesion depth after treatment followed by the 16-h acid attack (*P* < 0.05).

Surface microhardness of enamel specimens is plotted in **Figure 9A**. Compared with untreated control, CAP-treated enamels (3 min) displayed less percentage of surface microhardness loss after 16 h of cariogenic challenge.

### 3.8 Micro-CT analysis

**Figure 9B** shows the lesion depth of enamel under micro-CT analysis. Results from micro-CT analysis showed the similar trend as surface microhardness loss. CAP 1- or 3-min treatment decreased enamel demineralization depth than non-treatment control (bacteria control). CAP demonstrated a protective effect in enamel demineralization, which was better than UV under the same tested condition.

## 4 Discussion

The present study investigated a CAP device, which is designed to be usable in the human oral environment. The aim of this study is the application of a CAP device in the intraoral environment, where the stability of the discharge is a prerequisite for safety. The device used a double-ring discharge structure with quartz tubes as the dielectric material and argon as the discharge gas, which makes it easy to obtain plasma and provides discharge stability. The discharge temperature of the device was always below 40°, which is close to the temperature of the human oral cavity and does not cause burn damage to the soft tissues (Yazici et al., 2006). The voltametric characteristic curves and Lissajous of the device were examined separately using oscilloscope acquisition and Lissa-like graphs.

The effect of CAP action on biofilm growth and regrowth were obtained by MTT assay, a method by which oral plaque biofilm activity can be demonstrated and which is widely used in the study of dental antimicrobial materials (Han et al., 2017; Liang et al., 2018). The data showed that the immediate killing effect of CAP was more pronounced for monospecies biofilms and microcosm biofilms, with significant immediate inhibition of biofilm growth activity and lactate metabolism. Other studies provided strong evidence that defined configurations of atmospheric pressure air plasmas can achieve rapid and highly effective bacterial killing in a biofilm model (Modic et al., 2017), which tends to be close to this study. Furthermore, the biofilm still had the ability to be regrown; for monospecies biofilms and microcosm biofilms, different biological characteristics were shown during regrowth (Rostami et al., 2017). The MTT data showed no difference between the two biofilms after 24 and 48 h, and the activity of bacterial mitochondrial succinate dehydrogenase in biofilms was shown to approach normal levels after 24 h of CAP action.

The metabolic effect of CAP on oral plaque biofilms was demonstrated by the amount of lactic acid produced. However, the recovery of biofilm metabolic lactate differed from the MTT changes, with *S. mutans* biofilms and microcosm biofilms maintaining the effects of CAP on them in 8 h. Between 8 and 24 h, lactic acid production from *S. mutans* biofilms did not differ between groups, but there were differences between microbial biofilm groups, with lower acid production in the CAP-treated group than untreated controls. Previous studies have tended to focus more on the immediate effect of the antimicrobial material and less on the regrowth state of the biofilm after the action of the antimicrobial material. Therefore, the state of the biofilm after antibacterial treatment and the bacterial activity exhibited during regrowth are matters of concern (Han et al., 2019). Previously, oral disinfectants tended to leave residues in the mouth (Mikati et al., 2020), but the advent of CAP will make residue-free antimicrobial activity a reality. However, the CAP has a short time to exert its antimicrobial power, and the strong biofilm proliferation rebound after CAP stimulation is a drawback of the application, and how to improve this property will be the next step in this research.

Changes in the oral microbial communities in response to CAP were obtained by 16S ribosomal RNA assay. There is evidence that information on oral microbial community structure and function has great potential in predicting the occurrence and treatment prognosis of oral diseases (Bowen et al., 2018; Valm, 2019), such as caries. Moreover, CAP has been less studied in improving the structural and functional information of oral microbial communities. The data revealed that on the basis of the overall microbial load being suppressed, the bioactivity of *Lactococcus* spp. was significantly suppressed whereas that of *Streptococcus* spp. was less influenced, suggesting that external stimuli have a weaker effect on the bioactivity of *Streptococcus* spp. or that the self-protection mechanism of *Streptococcus* spp. is more complete. This is different from previous studies of antimicrobial materials. The possible reason for this is that CAP is a gaseous molecule and its effect is immediate, transient, or too little after the action to have a sustained effect (Wu et al., 2011), as previous antimicrobial materials were contact antimicrobial, which was different from this experiment.

CAP’s action on biofilms affecting enamel demineralization was obtained by surface hardness and cross-sectional micrographs. This study explored the future potential of plasma in the study of dental caries control through plaque microorganisms. In this part of the experiment plaque, biofilms were precultured on the enamel surface and the above biofilm culture process has been treated with this plasma to simulate the caries demineralization process *in vitro*. The CAP treatment leads to changes in the surface roughness and morphology of the hard tissues of the teeth and results in a reduction in the contact angle between water and glycol, as well as a significant reduction in carbon on the enamel surface, all of which have an effect on the mineralization and remineralization of the hard tissues of the teeth (Chen et al., 2013; Kermanshah et al., 2020).

This study provides an experimental basis for the development and application of CAP in the prevention and treatment of dental caries in the future. However, the present study was performed in *in vitro* models, which is relatively ideal and cannot mimic the complex environment of human oral cavity. Thus, an *in vivo* animal experiment is needed for the further application of CAP.

## 5 Conclusion

In summary, CAP can reduce the cariogenicity of dental biofilms and inhibit enamel demineralization. This study provides an experimental basis for the development and application of CAP in the prevention and treatment of caries in the future.

## Data availability statement

The data presented in the study are deposited in the NCBI SRA, accession number PRJNA938579.

## Ethics statement

All animal experimental procedures were approved by the Animal Ethics Committee of West China Hospital of Sichuan University, China (NO.WCHSIRB-D-2022-255).

## Author contributions

HZ: conceptualization; data curation; formal analysis; investigation; methodology; writing—original draft; writing—review and editing. XW: device assembly; data curation; formal analysis. ZL: device design; conceptualization; methodology. YW: data curation; funding acquisition; methodology. LZ: methodology; writing—review. YC: conceptualization; funding acquisition; resources; supervision; validation. QH: conceptualization; funding acquisition; resources; supervision; validation; writing—review and editing. All authors contributed to the article and approved the submitted version.
